# Integrated transcriptome catalog of *Tenualosa ilisha* as a resource for gene discovery and expression profiling

**DOI:** 10.1038/s41597-023-02132-z

**Published:** 2023-04-17

**Authors:** Md. Arko Ayon Chowdhury, Md. Rakibul Islam, Al Amin, Sadia Noor Mou, Kazi Newaz Ullah, Abdul Baten, Mohammad Shoyaib, Amin Ahsan Ali, Farhana Tasnim Chowdhury, Md. Lifat Rahi, Haseena Khan, M Ashraful Amin, Mohammad Riazul Islam

**Affiliations:** 1 Molecular Biology Laboratory, Department of Biochemistry and Molecular Biology, University of Dhaka, Dhaka, 1000 Bangladesh; 2 Center for Computational and Data Sciences (CCDS), Independent University, Bangladesh (IUB), Dhaka, Bangladesh; 3 Department of Zoology, Jagannath University, Dhaka, 1100 Bangladesh; 4 Institute of Precision Medicine and Bioinformatics, Sydney Local Health District, Royal Prince Alfred Hospital, Camperdown, Australia; 5 Institute of Information Technology (IIT), University of Dhaka, Dhaka, 1000 Bangladesh; 6 Fisheries and Marine Resource Technology (FMRT) Discipline, Khulna University, Khulna, 9208 Bangladesh

**Keywords:** Transcriptomics, Agriculture

## Abstract

The silver pride of Bangladesh, migratory shad, *Tenualosa ilisha* (Hilsa), makes the highest contribution to the total fish production of Bangladesh. Despite its noteworthy contribution, a well-annotated transcriptome data is not available. Here we report a transcriptomic catalog of Hilsa, constructed by assembling RNA-Seq reads from different tissues of the fish including brain, gill, kidney, liver, and muscle. Hilsa fish were collected from different aquatic habitats (fresh, brackish, and sea water) and the sequencing was performed in the next generation sequencing (NGS) platform. *De novo* assembly of the sequences obtained from 46 cDNA libraries revealed 462,085 transcript isoforms that were subsequently annotated using the Universal Protein Resource Knowledgebase (UniPortKB) as a reference. Starting from the sampling to final annotation, all the steps along with the workflow are reported here. This study will provide a significant resource for ongoing and future research on Hilsa for transcriptome based expression profiling and identification of candidate genes.

## Background

The migratory shad Hilsa (*Tenualosa ilisha*) is the national fish of Bangladesh and very famous for its unique taste and texture. It is an anadromous fish and spends most of its life cycle in the marine environment but mature individuals migrate to the upstream freshwater river systems for spawning. Post spawning, larvae grow up to the juvenile stage (locally known as *Jatka*) that perform downstream migration to the sea for further growth and maturation. This indicates that Hilsa regularly experiences broad spectrum salinity fluctuation throughout its life cycle. Therefore, this species possesses the capability to rapidly adapt to large scale salinity change including full strength seawater (salinity level 35.0%) to absolute freshwater (salinity level 0%) and vice versa^1^. Due to this sharp salinity fluctuation, Hilsa faces severe challenges including osmotic stress, differences in nutrient availability, changing immunity and disease susceptibility, hormonal imbalance, etc. Thus, Hilsa must display diverse adaptive strategies to rapidly cope with these biological changes during their migration pathways. Differential expression of a number of candidate genes is thought to be the key underlying mechanism to rapidly cope with these changing conditions.

**Fig. 1:**
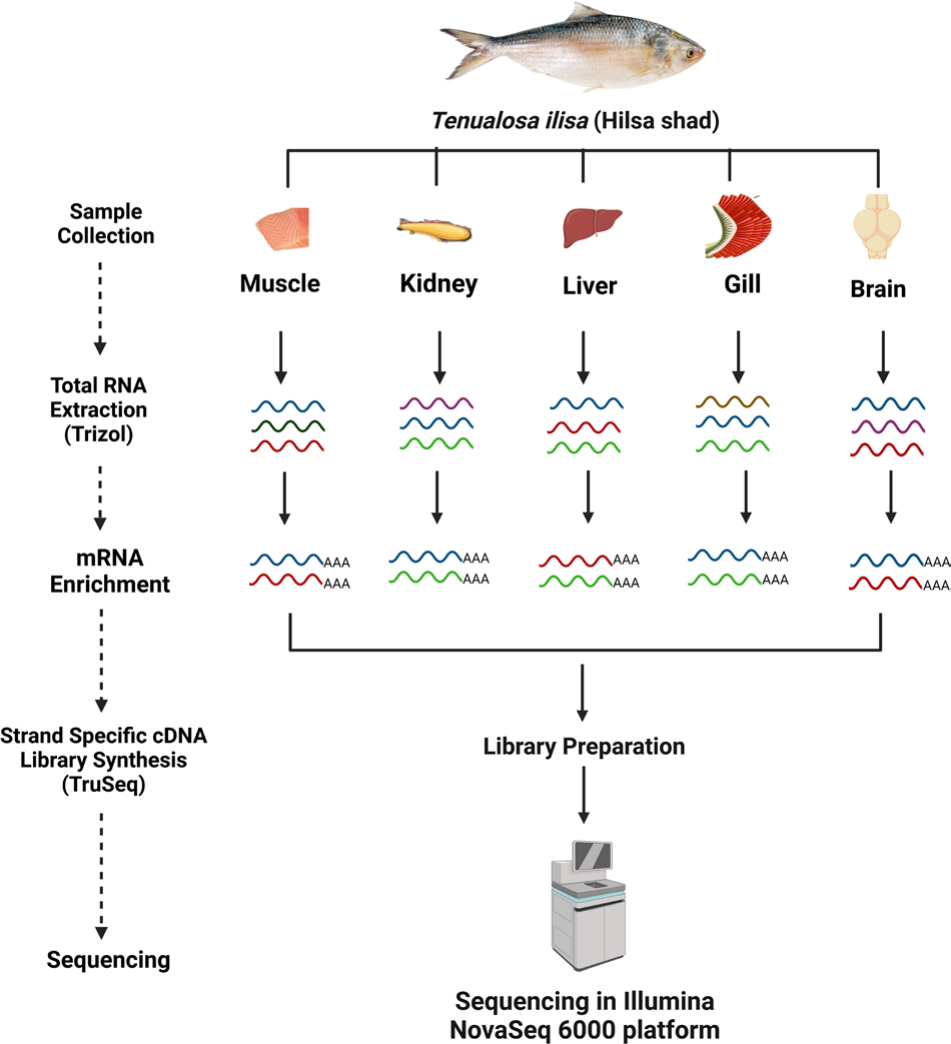
Workflow of RNA Sequencing of Hilsa tissue samples (created using Biorender.com).

**Fig. 2:**
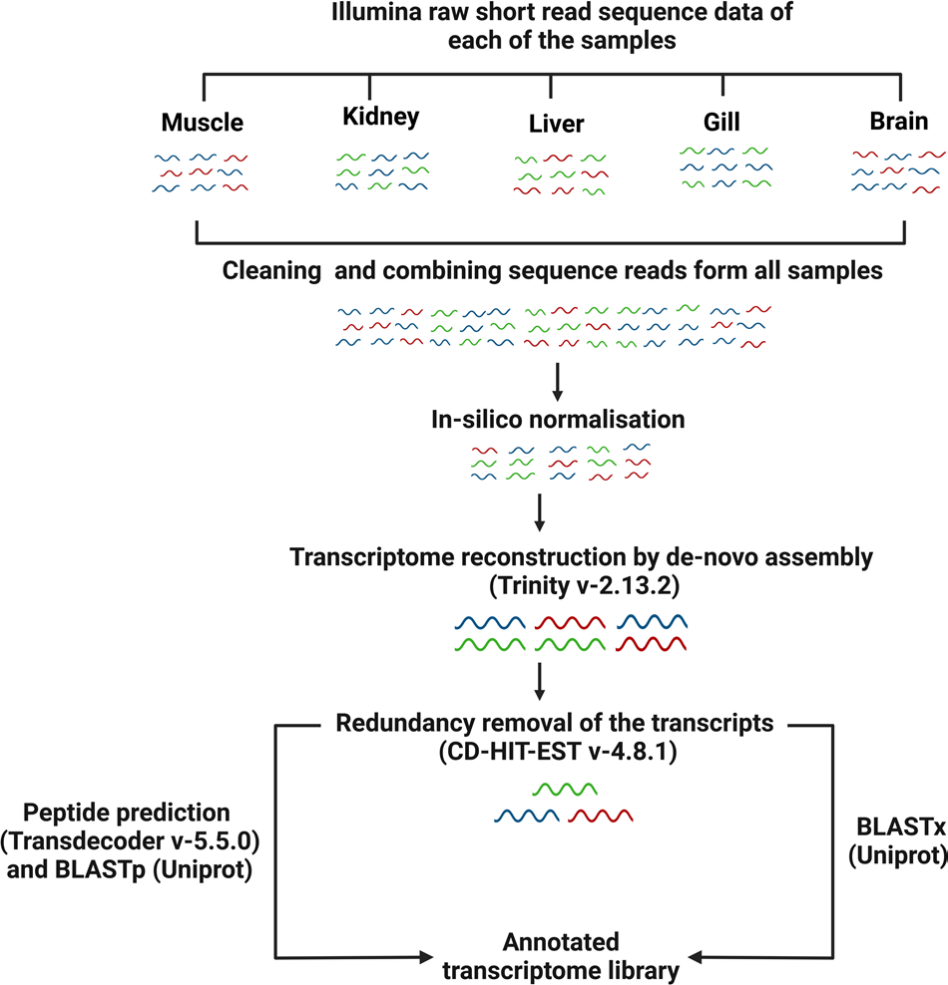
Bioinformatics workflow of transcript reconstruction and annotation (created using Biorender.com).

Hilsa is nutritionally enriched with high quality proteins, vitamins, minerals, polyunsaturated fatty acids (PUFA) and Omega-3 fatty acids^2^. It is considered to be the most important commercial and cultural species across the entire Indian sub-continent. In the financial year 2017–18, total 0.517 million Metric Ton of Hilsa was harvested in Bangladesh alone, which constitutes 12% of total national fish production^3^. Since, Hilsa is of central importance as a primary fisheries species in Bangladesh, more in-depth molecular work is needed to understand their physiology and adaptation mechanism in both marine and fresh aquatic environments. At present, 3 draft genome assemblies of Hilsa are publicly available^4–6^. The publicly available *de novo* transcriptome assemblies for Hilsa are limited to either muscle or liver tissues^7,8^. In a recent study, tissue-specific diversity of the alpha-2-macroglobulin splice isoforms in liver, gill, testes, and ovary have been reported but no annotated transcripts were made publicly available^9^. Here we report an annotated transcriptome catalog of Hilsa using five different tissues (gill, kidney, liver, brain and muscle) with sequential workflow (Figs. 1,2). These five tissues were selected based on diverse functional roles of the respective tissues. Brain and liver constitute most of the major regulatory functions of body and thus most of the genes are expressed in these two tissues. Different genes responsible for inhabiting heterogeneous environmental conditions are expressed in the gill and kidney tissues. Genes associated with growth, developmental processes, metabolic and physiological activities are normally expressed in muscle tissue. Therefore, these five tissues provide ideal samples for capturing almost all of the expressed genes in a fish. This annotated *de novo* transcriptome assembly from *in silico* normalised RNA-Seq reads is expected to serve as a complementary resource and reference gene sets, which will accelerate Hilsa research on gene discovery and genome annotation using experimental evidence, gene expression profiling, etc^10^.

In-depth sequencing (at least 80 million paired end reads per sample) has enhanced the chance of including the low abundant transcripts in the count^11^.

## Methods

### Sample collection

**Fig. 3:**
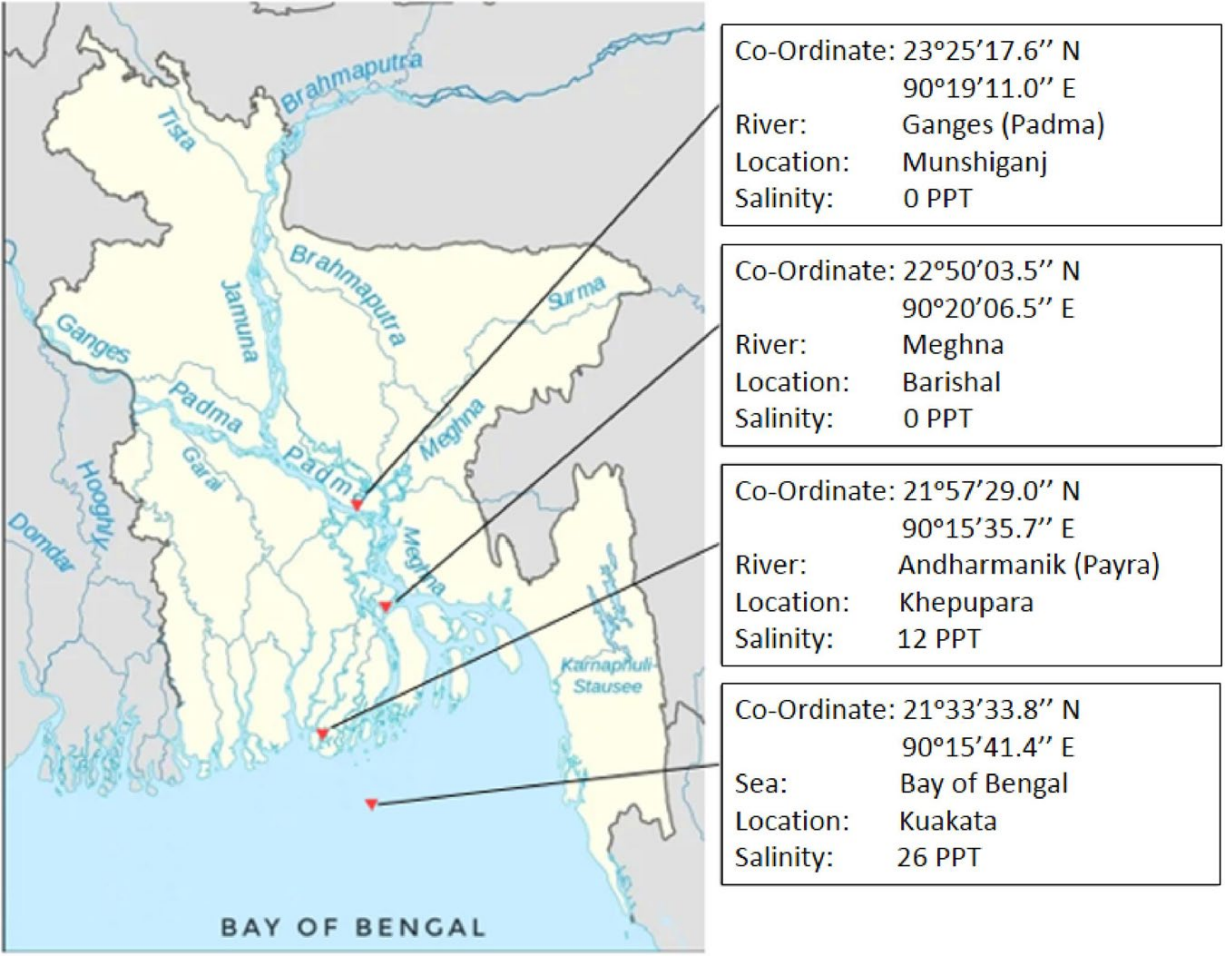
Map showing the sampling sites of the Hilsa fish collection (geographic co-ordinates, place and name of the river systems with respective salinity levels).

At least, four live Hilsa samples were collected from each sampling sites using seine net with the help of local fishermen (Fig. 3). Live fish samples were euthanized using dry ice (1:1 wt/wt) and immediately dissected in the field to obtain fresh tissue samples. Gill, kidney, liver, brain and muscle tissues were dissected and immediately preserved in RNAlater. The preserved Hilsa tissue samples were brought to the laboratory and maintained at −80 °C for subsequent use. All the required paper works for animal ethics clearance and field work were approved by authority prior to the starting of this study (Ref. No.: KUAEC-2021/09/20).

### RNA extraction

RNA was isolated from the RNAlater preserved tissue samples using TRIzol® reagent (ThermoFisher Scientific, USA)^12^. The manufacturer’s protocol was optimized with slight modifications. Tissue samples taken from RNAlater were washed with ice-cold DEPC-treated water and transferred immediately in mortars containing liquid nitrogen. Tissue samples were crushed into fine powder using pestles. Finely crushed tissue samples were subsequently transferred to 1 mL of ice-cold TRIzol solution and maintained on ice for 10 minutes. After a short vortex and centrifugation (16,000 × g for 15 minutes at 4 °C temperature), the supernatant (pink aqueous solution) was transferred to another microcentrifuge tube. 300 μL of chloroform was added to each tube and mixed well by inverting. The mixture was incubated on ice for 10 minutes prior to centrifugation at 16,000 × g. After centrifugation, 450 μL of the aqueous layer was pooled carefully and transferred to fresh microcentrifuge tubes without disturbing the other layer. 45 μL of 3M sodium-acetate (pH~5.5) and 495 μL of isopropanol were added in the tubes and incubated overnight at −20 °C.

The supernatant was discarded after centrifugation at 16,000 × g for 15 minutes at 4 °C. A slightly white clear pellet was formed at the bottom of each tube. 1 mL of 75% ethanol was added to each tube for washing and then centrifuged (16,000 × g for 15 minutes at 4 °C). The supernatant was discarded and pellets were air dried for 20 minutes and resuspended in 30 μL of DEPC treated water. RNA concentration was measured using a Nanodrop One UV-vis spectrophotometer. Integrity of the extracted RNA was checked using agarose gel electrophoresis and Agilent Technologies 2200 TapeStation RNA ScreenTape^13^. Only the high quality RNA samples were sent to Macrogen Inc, SouthKorea for subsequent steps including library preparation and sequencing.

### cDNA Library preparation and sequencing

Removal of contaminating genomic DNA by DNase-l treatment, mRNA isolation from total RNA samples, cDNA library preparation and quality assessment were performed at Macrogen Inc, South Korea. TruSeq stranded mRNA library (Illumina, San Diego, USA) preparation kit was used to prepare Hilsa cDNA libraries following the manufacturer’s protocol. Constructed cDNA libraries were then assessed for quality using Bioanalyzer. Equimolar quantities of each good quality cDNA libraries (Table 1) were used for sequencing in the Illumina based platform, NovaSeq 6000.

**Table 1:** Number of source specific cDNA libraries used in sequencing.

Source/Organ	Brain	Muscle	Gill	Liver	Kidney
Padma River (Fresh water)	3	2	1	1	—
Meghna River (Fresh water)	3	3	3	3	3
Payra River (Brackish water)	2	3	3	1	3
Bay of Bengal (Sea water)	3	3	2	2	2

### *In silico* normalisation and *de novo* assembly and dataset annotation

The software package FastP (version 0.12.4) was used to remove extraneous (first 12 bases) sequences and a default sliding window size 4 with a mean quality score below Q20 was trimmed prior to *de novo* assembly^14^. After filtering the initial 4,560,499,900 reads, total 4,319,117,376 high quality sequencing reads were remained. The quality of all the trimmed reads was evaluated individually using FastQC (https://www.bioinformatics.babraham.ac.uk/projects/fastqc/) and aggregated using MultiQC^15^. Clean paired-end reads from 46 samples were first normalised *in silico* and further assembled into 527,646 transcript isoforms using Trinity (version-2.13.2)^16,17^. GC content of the transcriptome was 44.56%, median and average transcript lengths were 358 and 681.61 bases respectively. Redundant transcripts were removed by clustering the sequences using the CD-HIT-EST program with a default (0.95) similarity match threshold that resulted in 462,456 transcript isoforms^18^. Subsequently, the transcript sequences were submitted to NCBI Transcriptome Shotgun Assembly (TSA) Database. NCBI screened the transcriptome assembly for foreign contaminating transcripts. The contaminating transcripts were removed and final number of transcripts were 462,085. Coding regions within the transcripts were predicted and extracted using the TransDecoder (version-5.5.0) (https://github.com/TransDecoder/TransDecoder) tool with the default parameters. BLASTx and BLASTp programs (version-2.12.0+) were used for homology-based similarity search of the transcripts and predicted proteins against the latest UniProtKB protein database with a maximum e-value of 1e^−5^ ^19–21^. The BLAST results were integrated by Trinotate (http://trinotate.github.io).

## Data Records

Raw RNA-Sequence reads of different tissues of *Tenualosa ilisha* have been deposited in the NCBI Sequence Read Archive (SRA) database under the NCBI Bioproject (https://www.ncbi.nlm.nih.gov/bioproject/) with accession PRJNA850620^22^. Final set of transcripts have been submitted to NCBI Transcriptome Shotgun Assembly (TSA) database (https://www.ncbi.nlm.nih.gov/genbank/tsa/) under the accession no GKAU00000000^23^. Raw assembly data, annotation file and Transdecoder predicted peptide files have been deposited in Figshare^24^.

## Technical Validation

**Fig. 4:**
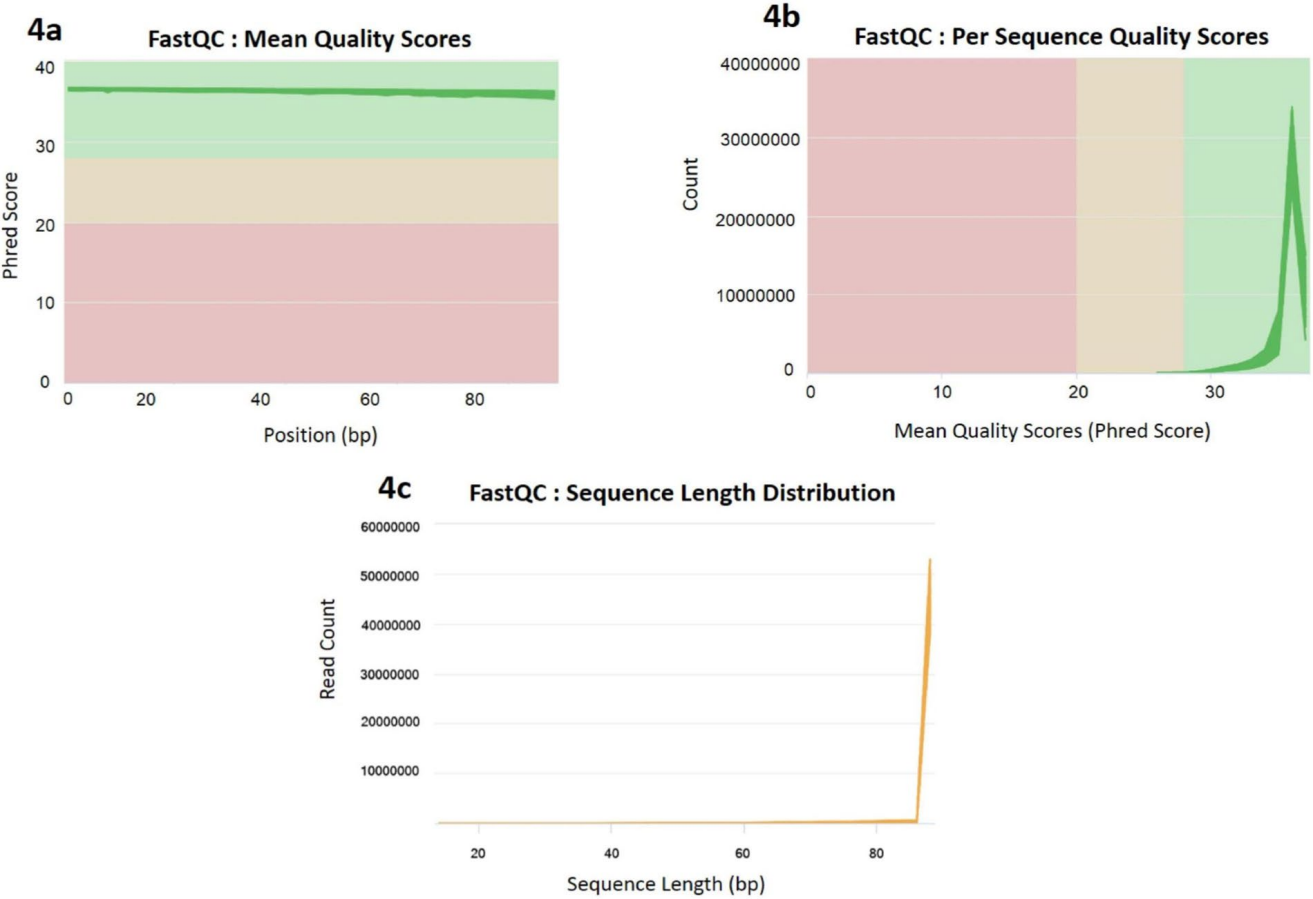
Sequence quality assessment metrics by MultiQC of filtered and trimmed RNA-Seq data of all the samples. (**a**) The mean quality scores across each base position, (**b**) Number of reads with average quality scores, (**c**) The distribution of fragment sizes/read lengths.

**Fig. 5:**
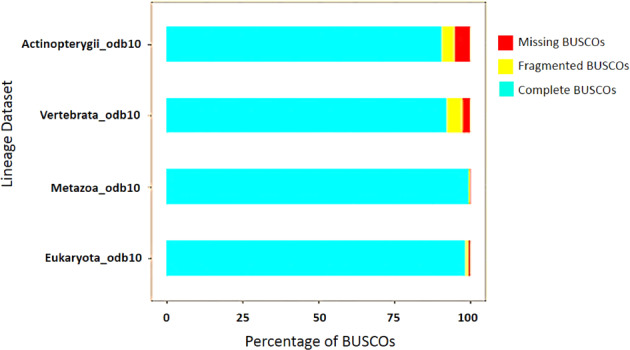
Assessment of the Hilsa transcriptome completeness by Benchmarking Universal Single-Copy Orthologs (BUSCO).

Samples with low quality RNA and cDNA library were removed from the transcriptome catalog. From raw reads to final annotation, the quality of data was scrutinised at several checkpoints. The quality of the raw and trimmed sequence reads was assessed in terms of sequence quality, presence of adaptors, GC content, overrepresented k-mers using FastQC (http://www.bioinformatics.babraham.ac.uk/projects/fastqc). Subsequently QC of all the samples were aggregated and analysed altogether utilizing MultiQC (Fig. 4). An appropriate reference genome of Hilsa with chromosomal information is not available^4–6^. Thus, transcriptome was reconstructed from the RNA-Seq data using the Trinity *de novo* RNA-Seq assembler which outperforms others in terms of overall reads representation of the assembly, completeness, mismatch and mis-assembly, etc^25^. The transcriptome data were subjected to BUSCO (Benchmarking Universal Single-Copy Orthologs) analysis to examine their completeness^26,27^. The analysis performed on transcriptome module against eukaryota_odb10, metazoa_odb10, vertebrata_odb10 and actinopterygii_odb10 ortholog gene sets showed 98.40%, 99.60%, 92.30% and 90.70% of completeness respectively. Also, percentages of missing BUSCOs for eukaryota_odb10, metazoa_odb10, vertebrata_odb10 and actinopterygii_odb10 datasets were found to be 0.4%, 0.2%, 2.5% and 5.0% respectively (Fig. 5)^28^.

Both the BLASTp and BLASTx programs for annotation were used with a stringent e-value cut-off of 1e^−5^ against the UniprotKB Protein Database. The workflow and the links of the deposited data are provided. These datasets expand the indispensable transcriptomic resources for further research on Hilsa functional genomics, gene characterisation and expression profiling etc.

## Data Availability

**Fastp (version 0.12.4)** *fastp -i Read_1.fastq.gz -f 12 -o Read_1_fastp_trim.fastq -I Read_2.fastq.gz -F 12 -O Read_2_fastp_trim.fastq* **Trinity (version 2.13.2)** *Trinity–seqType fq–max_memory 96* *G–samples_file Hilsa_samples–SS_lib_type RF–CPU 20–no_bowtie* Hilsa_samples file is provided in Figshare repository^23^. **CD-HIT (version 4.8.1)** *cd-hit-est –I Hilsa_RNA_Trinity.fasta –o Hilsa_TSA.fasta –T 20 –M 1400 –c 0.95* **TransDecoder (version 5.5.0)** *TransDecoder.LongOrfs -t Hilsa_TSA_v2.fasta* Then, *TransDecoder.Predict -t HILSA_TSA_v2.fasta–retain_blastp_hits Hilsa_peptides_BLASTp.txt* **BLAST (version 2.12.0+)** **Database Preparation:** *makeblastdb –in uniprot_sprot.fasta -dbtype prot -parse_seqids -out uniprot_sprot_fasta.db* **BLASTp RUN-1:** *blastp -query Hilsa_peptide_transdecoder.fasta -db uniprot_sprot_fasta.db -outfmt 6* *-max_target_seqs 1 -num_threads 16 -evalue 1e-5 -out Hilsa_peptides_BLASTp.txt* The output file ‘Hilsa_peptides_BLASTp.txt’ was used to run ‘TransDecoder.Predict’ program. **BLASTp RUN-2:** *blastp -query HILSA_TSA_v2.fasta.transdecoder. fasta -db uniprot_sprot_fasta.db -outfmt 6 -max_target_seqs 1 -num_threads 16 -evalue 1e-5 -out Hilsa_final_BLASTp.txt* **BLASTx RUN:** *blastx -db uniprot_sprot_fasta.db -query Hilsa_TSA.fasta -max_target_seqs 1 -outfmt 6* *-num_threads 16 -evalue 1e-5* > *Hilsa_Transcripts_Blastx.txt* **Trinotate** *Build_Trinotate_Boilerplate_SQLite_db.pl Trinotate* Then, *Trinotate Trinotate.sqlite init–gene_trans_map Hilsa_TSA_v2.fasta.gene_trans_map –* *–transcript_fasta Hilsa_TSA_v2.fasta–transdecoder_pep Hilsa_peptide_transdecoder.fasta* **Loading BLASTx and BLASTp results** *Trinotate Trinotate.sqlite LOAD_swissprot_blastx Hilsa_Transcripts_Blastx.txt* And then, *Trinotate Trinotate.sqlite LOAD_swissprot_blastp Hilsa_ final _BLASTp.txt* **Generating Annotation Report** *Trinotate Trinotate.sqlite report* > *Annotation_report.xls*
